# Spontaneous Bacterial Peritonitis Caused by *Bordetella hinzii*

**DOI:** 10.3201/eid2711.211428

**Published:** 2021-11

**Authors:** Grace C. Wang, Miranda J. Wallace, Gayathri Krishnan, Patrick D. Olson, Abigail L. Carlson, Gautam Dantas, James M. Fleckenstein

**Affiliations:** Saint Louis University School of Medicine, St. Louis, Missouri, USA (G.C. Wang);; Washington University School of Medicine, St. Louis (M.J. Wallace, G. Krishnan, P.D. Olson, A.L. Carlson, G. Dantas, J.M. Fleckenstein);; St. Louis Veterans Affairs Health Care System, St. Louis (A.L. Carlson, J.M. Fleckenstein)

**Keywords:** Bordetella hinzii, spontaneous bacterial peritonitis, whole genome sequencing, bacteria, bacteremia, coccobacilli, Missouri, United States

## Abstract

Although *Bordetella hinzii* coccobacilli is most commonly identified in respiratory tracts of birds and rodents, this organism has occasionally been isolated in human infections. We describe a case of *B. hinzii* spontaneous bacterial peritonitis in Missouri, USA. Whole-genome sequencing of blood and peritoneal fluid isolates confirmed *B. hinzii* infection.

*Bordetella hinzii* is a gram-negative aerobic coccobacilli respiratory pathogen in poultry (*1*) and rodents (*2*). Human infections are rare but occur in immunocompromised persons (*3*) or upon exposure to infected animals. Most reported human infections are pulmonary; however, other manifestations include cholangitis (*4*) and periaortic abscess (*5*). We report a case of *B. hinzii* spontaneous bacterial peritonitis (SBP) complicated by bacteremia.

A 71-year-old man with alcoholism, hepatitis C, and decompensated cirrhosis, on day 28 of a 28-day regimen of intravenous vancomycin for *Streptococcus salivarius* bacteremia and SBP, underwent outpatient paracentesis. After paracentesis (7.8 L of fluid removed), the patient experienced hypotension and orthostasis, which resolved after intravenous albumin, and returned home. He later sought care at an emergency department for weakness, abdominal pain, hypotension (68/42 mm Hg), and tachycardia (heart rate 130 beats/min).

Despite intravenous fluid and albumin (1.5 g/kg) resuscitation, daptomycin, and piperacillin/azobactam, the patient experienced septic shock and hepatorenal syndrome, necessitating pressors. We substituted meropenem for piperacillin/tazobactam. On day 3, we discontinued daptomycin; administered albumin (1 g/kg); and initiated octreotide, midodrine, and rifaximin. We discontinued pressors on day 4. The patient improved clinically and his SBP resolved, as indicated by results of serial peritoneal fluid studies. On day 12, we replaced meropenem with ertapenem. After discharge (day 15), the patient completed 2 weeks of ertapenem, followed by daily trimethoprim/sulfamethoxazole prophylaxis.

Further investigation revealed the patient had a dog with cough and a cat with gastroenteritis, and neither pet was receiving veterinary care. His wife maintained several birdfeeders, but she had no symptoms. Five months after seeking care, the patient died after cardiac arrest and transjugular intrahepatic portosystemic shunt occlusion.

Initial ascites fluid studies revealed 1,673 leukocytes with 80% segmented neutrophils. Peritoneal fluid and blood cultures from day 1 of hospitalization had tiny oxidase-positive, indole-negative, gram-negative rods that grew on blood and chocolate agars but not MacConkey or CNA agars. After tentatively identifying the organism as a *Bordetella* species, we performed limited antimicrobial susceptibility testing for levofloxacin (1.5 μg/mL), ertapenem (0.19 μg/mL), meropenem (sensitive), and trimethoprim/sulfamethoxazole (0.004 μg/mL). The Missouri State Public Health Laboratory (Jefferson City, MO, USA) later confirmed identification as *B. hinzii* by using matrix-assisted laser desorption/ionization time-of-flight mass spectrometry. 

Blood and peritoneal fluid isolates underwent whole-genome sequencing (WGS) to confirm identification. We suspended isolate plate scrapes in Luria–Bertani broth with 15% glycerol and stored them at –80°C. After thawing 250–500 μL of each suspension, we extracted genomic DNA by using the QIAamp BiOstic Bacteremia DNA Kit (QIAGEN, https://www.qiagen.com). We used 0.5 ng of genomic DNA to prepare sequencing libraries with the Nextera XT DNA Library Preparation Kit (Illumina, https://www.illumina.com). We pooled and sequenced libraries on the NovaSeq 6000 platform (Illumina) to obtain ≈5 million 2 × 150 bp reads. We used Trimmomatic 38.0 (https://github.com/timflutre/trimmomatic) to demultiplex the reads and remove adaptors. We removed contaminating reads by using Deconseq4.3 (http://deconseq.sourceforge.net) and repaired disordered reads by using BBTools Repair (BBTools 38.26; https://jgi.doe.gov/data-and-tools/bbtools). We assembled de novo genomes by using the Unicycler 0.4.7 Illumina-only assembly process and then annotated by using Prokka 1.14.5 (https://github.com/tseemann/prokka). We determined assembly quality by using QUAST 4.5 (http://quast.sourceforge.net) and checkM 1.0.13 (https://github.com/Ecogenomics/CheckM).

We performed pairwise average nucleotide identity between the isolates and deposited *Bordetella* genomes by using pyani (https://github.com/widdowquinn/pyani). We performed core-genome alignments by using roary 3.12.0 (https://sanger-pathogens.github.io/Roary) and then generated approximate maximum-likelihood trees on the basis of the roary alignment file by using FastTree 2.1.7 (http://www.microbesonline.org/fasttree). We identified single-nucleotide polymorphisms (SNPs) by using Snippy 4.4.3 (https://anaconda.org/bioconda/snippy/files) and antimicrobial-resistance genes by using AMRFinder 3.8.4 (https://github.com/ncbi/amr). We deposited raw sequence data and genomic assemblies to the National Center for Biotechnology Information (BioProject no. PRJNA706405).

We performed Illumina short-read WGS on 6 putative *B. hinzii* isolates recovered from peritoneal fluid and blood cultures from day 1 and a peritoneal fluid culture from paracentesis on day 5. Altogether, the isolate assemblies had an average length of 4.8 Mbp (range 4.70–4.84 Mbp) and GC content of ≈67.2%, reflective of published *B. hinzii* genomes (*6*). We built a neighbor-joining phylogenetic tree by using a core-genome alignment of the isolates with publicly available *Bordetella* genomes. The isolates formed a clade with *B. hinzii* genomes, including a type that was distinct from other *Bordetella* species. Pairwise average nucleotide identity analysis showed the isolates meet the species-level threshold (>96%) (*7*) exclusively with genomes originating from *B. hinzii* (Figure). SNP analyses within the 6 isolates suggested they were clonal because <2 SNPs (all nonsynonymous) were found between each strain pair, further confirming the clinical laboratory indications that the isolates are *B. hinzii* and that organisms recovered from peritoneal sites and blood originated from the same source. In addition, we identified a putative novel β-lactamase gene with 51% identity to the class-A LRA-1 β-lactamase (Comprehensive Antibiotic Resistance Database [https://card.mcmaster.ca/home]; accession no. ARO:3002482). This gene is likely endogenous because it appeared in all available *B. hinzii* assemblies.

**Figure:**
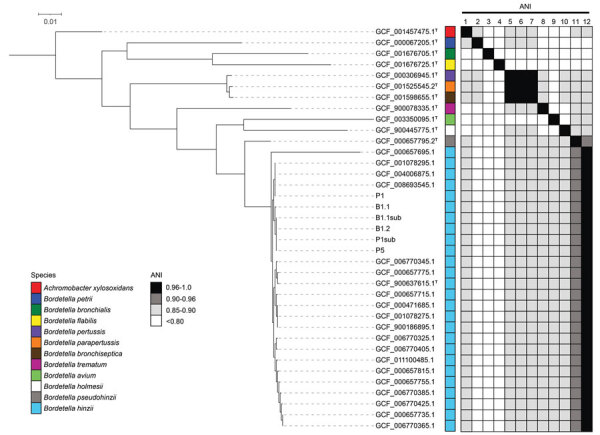
Comparative genomic analyses of *Bordetella hinzii* isolates from a patient in Missouri, USA, with type and nontype *Bordetella* assemblies. After core-genome alignment (58 total core genes), a neighbor-joining phylogenetic tree rooted with *Achromobacter xylosoxidans* as the outgroup demonstrates the isolates from this study cluster with other previously deposited *B. hinzii* genomes. Pairwise ANI was performed against type assemblies. The isolates in this study meet the ANI threshold (>0.96%) for species-level identity with *B. hinzii* type assembly GCF_900637615.1 (*7*). Isolates were recovered from peritoneal fluid cultures collected at day 1 and day 5 (P1 and P5, respectively; P2sub is a subculture of P1). Blood isolates were recovered from blood cultures collected on day 1 (B1.1 and B1.2; B1.1sub is a subculture of B1.1). As previously observed (*8*), the type genomes for *B. pertussis*, *B. parapertussis*, and *B. bronchiseptica* represent an instance of previously established, distinct species that exceed the species-level ANI threshold relative to each other. ^T^ indicates assemblies generated from type material. Type assemblies are numbered 1–12 on vertical axes as follows: 1, GCF_001457475.1; 2, GCF_000067205.1; 3, GCF_001676705.1; 4, GCF_001676725.1; 5, GCF_000306945.1; 6, GCF_001525545.2; 7, GCF_001598655.1; 8, GCF_900078335.1; 9, GCF_003350095.1; 10, GCF_900445775.1; 11, GCF_000657795.2; 12, GCF_900637615.1. ANI, average nucleotide identity.

 In summary, WGS of blood and peritoneal fluid isolates confirmed a set of clonal *B. hinzii* isolates from both tissue types from this patient. Our findings provide compelling evidence for serious human infection caused by this organism.
